# T Cells in Multisystem Inflammatory Syndrome in Children (MIS-C) Have a Predominant CD4+ T Helper Response to SARS-CoV-2 Peptides and Numerous Virus-Specific CD4− CD8− Double-Negative T Cells

**DOI:** 10.3390/ijms23137219

**Published:** 2022-06-29

**Authors:** Li-En Hsieh, Jaeyoon Song, Alba Grifoni, Chisato Shimizu, Adriana H. Tremoulet, Kirsten B. Dummer, Jane C. Burns, Alessandro Sette, Alessandra Franco

**Affiliations:** 1 Department of Pediatrics, School of Medicine, University of California San Diego, La Jolla, CA 92093, USA; l2hsieh@health.ucsd.edu (L.-E.H.); jac037@health.ucsd.edu (J.S.); c1shimizu@health.ucsd.edu (C.S.); atremoulet@health.ucsd.edu (A.H.T.); kdummer@health.ucsd.edu (K.B.D.); jcburns@health.ucsd.edu (J.C.B.); 2 Division of Vaccine Discovery, La Jolla Institute for Immunology, La Jolla, CA 92037, USA; agrifoni@lji.org (A.G.); alex@lji.org (A.S.); 3 Rady Children’s Hospital, 3020 Children’s Way, San Diego, CA 92123, USA; 4 Department of Medicine, University of California, San Diego (UCSD), La Jolla, CA 92037, USA

**Keywords:** MIS-C, SARS-CoV-2, T cells, CD4− CD8− double-negative T cells, T cell receptor Vβ21.3

## Abstract

We studied SARS-CoV-2-specific T cell responses in 22 subacute MIS-C children enrolled in 2021 and 2022 using peptide pools derived from SARS-CoV-2 spike or nonspike proteins. CD4+ and CD8+ SARS-CoV-2-specific T cells were detected in 5 subjects, CD4+ T helper (Th) responses alone were detected in 12 subjects, and CD8+ cytotoxic T cell (CTL) responses alone were documented in 1 subject. Notably, a sizeable subpopulation of CD4− CD8− double-negative (DN) T cells out of total CD3+ T cells was observed in MIS-C (median: 14.5%; IQR 8.65–25.3) and recognized SARS-CoV-2 peptides. T cells bearing the Vβ21.3 T cell receptor (TcRs), previously reported as pathogenic in the context of MIS-C, were detected in high frequencies, namely, in 2.8% and 3.9% of the CD4+ and CD8+ T cells, respectively. However, Vβ21.3 CD8+ T cells that responded to SARS-CoV-2 peptides were detected in only a single subject, suggesting recognition of nonviral antigens in the majority of subjects. Subjects studied 6–14 months after MIS-C showed T cell epitope spreading, meaning the activation of T cells that recognize more SARS-CoV-2 peptides following the initial expansion of T cells that see immunodominant epitopes. For example, subjects that did not recognize nonspike proteins in the subacute phase of MIS-C showed good Th response to nonspike peptides, and/or CD8+ T cell responses not appreciable before arose over time and could be detected in the 6–14 months’ follow-up. The magnitude of the Th and CTL responses also increased over time. In summary, patients with MIS-C associated with acute lymphopenia, a classical feature of MIS-C, showed a physiological response to the virus with a prominent role for virus-specific DN T cells.

## 1. Introduction

A unique feature of the SARS-CoV-2 pandemic has been the emergence of a new syndrome in children exposed to SARS-CoV-2 who present 4 to 6 weeks later with fever and severe systemic inflammation requiring admission to an intensive care unit [1]. Multisystem inflammatory syndrome in children (MIS-C) [2,3] has been clinically well defined, but the pathogenesis is still debated [4,5,6].

In COVID-19, T cells play an important role in controlling SARS-CoV-2 infection [7,8]. Coordinated CD4+ T helper (Th) and CD8+ cytotoxic T cell (CTL) responses were associated with reduced disease severity in SARS-CoV-2-infected healthy convalescents, indicating a clear role for early T cell responses, in concert with antibodies, in the protective immunity to SARS-CoV-2 [8,9].

MIS-C subjects studied by our group in 2020 at the beginning of the COVID-19 pandemic mounted a T cell response to SARS-CoV-2, comparable to adult and pediatric patients recovering from COVID-19 [10]. In contrast, other investigators have postulated a spike protein epitope that serves as a superantigen and leads to the expansion of proinflammatory CD8+ T cells that express the Vβ21.3 T cell receptor (TcRs) and gut permeability in exposing autoantigens [11,12,13].

Here, we studied SARS-CoV-2-specific T cell responses, including the development of T cell memory, the expansion of Vβ21.3 T cells in response to SARS-CoV-2 peptides, and the innate immune phenotype in children with MIS-C enrolled later in the pandemic (2021–2022) not previously reported. These subjects were presumably exposed to different SARS-CoV-2 variants compared with the MIS-C subjects studied in the first year of the COVID-19 pandemic [10]. We included in our study an analysis of the T cell responses in the previously studied MIS-C subjects at their follow-up visit 6–14 months later.

## 2. Results

### 2.1. Detection of SARS-CoV-2 CD4+ and CD8+ T Cell Responses in MIS-C Subjects

We enrolled 22 MIS-C subjects (Table 1) to study the T cell response to spike and nonspike SARS-CoV-2 peptide pools.

Peripheral blood mononuclear cells (PBMCs) were stimulated in vitro with different peptide megapools tailored to capture T helper (Th) CD4+ T cell responses and cytotoxic CD8+ T cell (CTL) responses. CD4+ T cell responses were evaluated using the AIM assay by measuring by flow cytometry the expression of two costimulatory molecules, the tumor necrosis factor receptor superfamily member 4, also known as CD134 or OX40 receptor, and the tumor necrosis factor ligand superfamily member 9, also known as 4-1BB, 24 h after incubation of PBMC cultures with peptide megapools. CD8+ T cell responses were evaluated by measuring the expression of 4-1BB and CD69 24 h after incubation of PBMC cultures with peptide megapools.

**Figure 1:**
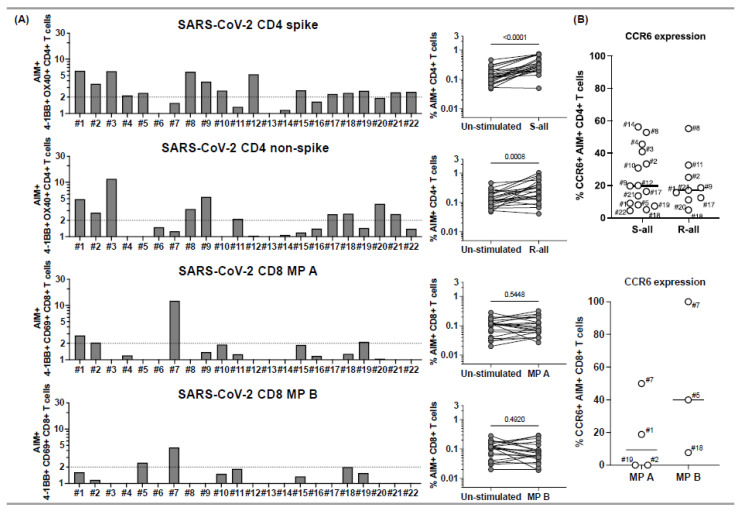
CD4+ and CD8+ T cell responses to peptide megapools derived from SARS-CoV-2 and their CCR6 expression in subacute MIS-C subjects. PBMCs from 22 subacute MIS-C subjects were separated from heparinized whole blood samples using Ficoll density gradient and stimulated with peptide megapools derived from SARS-CoV-2: CD4 spike (253 epitopes), CD4 nonspike (221 epitopes), CD8 MP A (314 epitopes), or CD8 MP B (314 epitopes). Cell preparations were collected 24 h after stimulation, followed by staining using monoclonal antibodies, to study the T cell activation in response to peptide megapool stimulations and the CCR6 expression on SARS-CoV-2-specific CD4+ and CD8+ T cells by flow cytometry. The T cell responses to the peptide megapools were calculated by dividing the percentage of activated T cells under different stimulatory conditions by the percentage of activated T cells in the unstimulated vehicle controls and were shown as stimulation index (SI). An SI ≥ 2 was considered a positive T cell response to the peptide megapool. (**A**) CD4+ and CD8+ T cell responses in responses to SARS-CoV-2 peptide megapools in individual subacute MIS-C subject. CD4+ T cell activation was defined by the coexpression of 4-1BB and OX40 (AIM+) gated under CD4+ T cells. CD8+ T cell activation was defined by the coexpression of 4-1BB and CD69 (AIM+) gated under CD8+ T cells. Five (1, 2, 5, 18, and 19) of the 22 subjects showed coordinate CD4+ and CD8+ T cell responses to SARS-CoV-2 CD4 and CD8 peptide megapools. Twelve subjects (3, 4, 8–12, 15, 17, and 20–22) of the 22 studied showed T cell responses to SARS-CoV-2 CD4 peptide megapools, and 1 (7) of the 22 subjects showed T cell responses to only SARS-CoV-2 CD8 peptide megapools. The T cell responses to SARS-CoV-2 CD4 and CD8 peptide megapools were not detected in 4 subjects (6, 13, 14, and 16) of the 22 subacute MIS-C subjects studied. Overall, CD4+ T cells from the MIS-C subjects showed more prevalent responses to SARS-CoV-2 CD4 spike (*p* < 0.0001) and nonspike (*p* = 0.0008) proteins, and CD8+ T cell responses were less prominent in the cohort (*p* > 0.05 for both SARS-CoV-2 CD8 megapool MP A and MP B). (**B**) CCR6 expression on SARS-CoV-2-specific CD4+ (top panel) and CD8+ (bottom panel) T cells. CCR6 expression can be found on both AIM+ CD4+ (median: 17.8%; Q1–Q3: 9.54–33.1%) and AIM+ CD8+ (median: 18.8%; Q1–Q3: 0–50%) T cells, although the expression of CCR6 on AIM+ CD8+ T cells varies between subjects. Each symbol represents data derived from an individual subject. Comparisons of the percentage of AIM+ T cells between unstimulated control and peptide megapool-stimulated cell cultures were tested by the Wilcoxon signed-rank test.

Of the 22 MIS-C subjects, 18 (82%) responded to SARS-CoV-2 megapools (Figure 1), and four had no response to any of the SARS-CoV-2 peptides (subjects 6, 13, 14, 16), a percentage that is similar to what we observed in a previous study during the first year of the COVID-19 pandemic [10]. Of the 18 who responded to the SARS-CoV-2 megapools, 5 subjects (28%), 1, 2, 5, 18, and 19, showed concurrent CD4+ T cell and CD8+ T cell responses to SARS-CoV-2 (Figure 1, panel A). An additional 5 subjects, (3, 8, 9, 17, and 21) showed only a CD4+ T cell response to both spike and nonspike peptide epitopes, while 5 subjects (4, 10, 12, 15, and 22) showed a CD4+ T cell response to spike proteins only, and 2 subjects (11 and 20) showed only a CD4+ T cell response to nonspike proteins (Figure 1, panel A). One subject, 7, had a CD8+ T cell response only (Figure 1, panel A).

Together, only 6/22 (27%) subjects had SARS-CoV-2-specific CTL in circulation. The CD4+ Th response was significantly higher than the CTL response in the MIS-C cohort (Figure 1, panel B). C-C chemokine receptor 6 (CCR6), which determines T cell homing to the endothelial and mucosal sites, was expressed with a different magnitude on AIM+ CD4+ and CD8+ T cells (Figure 1, panel C).

### 2.2. Expression of Memory Markers and Chemokine Receptors in SARS-CoV-2-Specific T Cells in MIS-C

**Figure 2:**
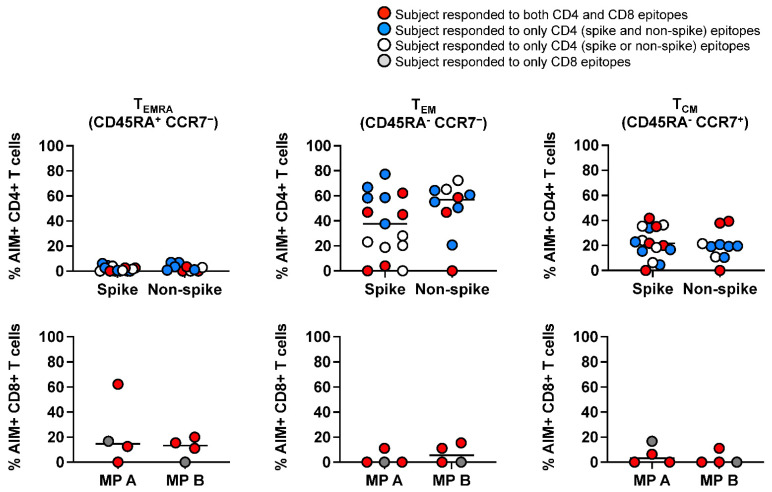
Memory phenotypes of SARS-CoV-2-specific, AIM+ CD4+, and CD8+ T cells in subacute MIS-C subjects. Memory phenotypes of SARS-CoV-2-specific AIM+ CD4+ (top panels) and CD8+ T cells (bottom panels) were studied 24 h after the peptide megapool stimulation. Each symbol shows the percentage of terminally differentiated effector T cells (TEMRA CD45RA+ CCR7−; left panels), effector memory T cells (TEM CD45RA− CCR7−; middle panels), and central memory T cells (TCM CD45RA− CCR7−; right panels) in the AIM+ CD4+ or CD8+ T cell populations. Red circles: subjects responding to both CD4 and CD8 epitopes (1, 2, 5, 18, and 19); blue circles: subjects responding to both CD4 spike and nonspike epitopes (3, 8, 9, 17, and 21); white circle: subject responding to either CD4 spike or nonspike epitopes (4, 10–12, 15, 20, and 22); gray circle: subject responding to only CD8 epitopes (7). Each symbol represents data derived from each individual subject. SARS-CoV-2-specific CD4+ T cells showed higher percentages of TEM and TCM phenotypes and lower percentages of TEMRA than SARS-CoV-2-specific CD8+ T cells.

Next, we characterized SARS-CoV-2-specific terminally differentiated effector T cells (TEMRA), effector memory T cells (TEM), and central memory T cells (TCM) by measuring the CD45RA and CCR7 expression on AIM+ T cells. SARS-CoV-2-specific CD4+ T cells were of the TEM and TCM subsets, and a low percentage of TEMRA cells were detected (Figure 2, upper panel). The T cell memory repertoire within SARS-CoV-2-specific CD8+ T cells was different. TEMRA cells were measurable in 6 subjects, and TEM and TCM cells were greater than 20% in 3 subjects and not detectable in 5 subjects despite the coordinated CD4+ T cell response (Figure 2, lower panels).

### 2.3. CD4− CD8− Double-Negative (DN) T Cells Are Numerous in Circulation and Respond to SARS-CoV-2 Peptides

**Figure 3:**
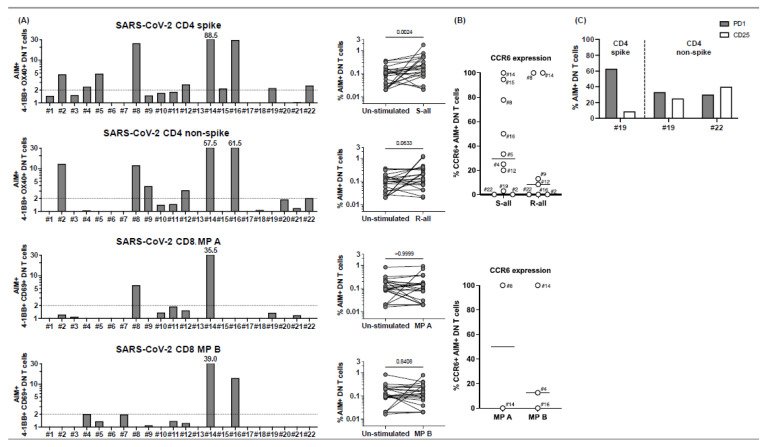
CD4− CD8− double-negative (DN) T cell responses to peptide megapools derived from SARS-CoV-2 in subacute MIS-C subjects. CD4− CD8− DN T cell responses were studied gated under CD3+ (T cells), followed by the gating of the CD4− CD8− population (see Supplementary Figure S1) in the 22 subacute MIS-C subjects. (**A**) DN T cell activation in response to SARS-CoV-2 peptide megapool stimulations. The percentages of 4-1BB+ OX40+ DN T cells were enumerated in unstimulated vehicle control, SARS-CoV-2 CD4 spike, and SARS-CoV-2 CD4 non-spike-stimulated cell cultures. In addition, the percentages of 4-1BB+ CD69+ DN T cells were enumerated in unstimulated vehicle control, SARS-CoV-2 CD8 MP A-, and MP B-stimulated cell cultures. SIs of AIM+ DN T cells from each individual subject are shown. Four subjects (4, 8, 14, and 16) of the 22 subjects showed DN T cell responses to both CD4 and CD8 peptide megapools. Seven subjects (2, 5, 9, 12, 15, 19, and 22) of the 22 MIS-C subjects showed DN T cell responses to only CD4 peptide megapools. No subject showed DN T cell responses to only CD8 peptide megapools, and 11 subjects (1, 3, 6, 7, 10, 11, 13, 17, 18, 20, and 21) of the 22 MIS-C subjects showed no measurable DN T cell response. (**B**) CCR6 expression on SARS-CoV-2-specific DN T cells. AIM+ DN T cells showed a different level of CCR6 expression from subject to subject. (**C**) PD1 and CD25 expression on SARS-CoV-2-specific DN T cells. PD1 and CD25 expressions were studied on 4 subjects (19–22). Subject 19 showed DN T cell responses to both CD4 spike and nonspike peptide megapools, and subject 22 showed DN T cell responses to a CD4 nonspike peptide megapool. Percentages of PD1+ (grey bars) and CD25+ (white bars) were enumerated under AIM+ DN T cells in the cell cultures, which showed a positive response to the peptide megapool. While 30.0–62.9% of the AIM+ DN T cells expressed PD1, 8.6–40.0% of the AIM+ DN T cells also showed CD25 expression. Each symbol represents data derived from each individual subject. Comparisons of the percentages of AIM+ DN T cells between unstimulated control and peptide megapool-stimulated cell cultures were tested by the Wilcoxon signed-rank test.

When we gated on CD3+ T cells in peptide-stimulated PBMC cultures to define AIM+ CD4+ and CD8+ T cells, we noted that a high percentage of the T cells (>40% in some subjects) were CD3+ but DN T cells that did not express CD4 or CD8 coreceptors (Supplementary Figure S1). To characterize this population, we first defined the ability of DN T cells to recognize the SARS-CoV-2 megapools determining the AIM+ T cells under stimulatory conditions. The results revealed that DN T cells are fully functional and respond to peptides with a greater stimulation index than canonical T cells (Figure 3, panel A). Subjects who showed a CD4+ T cell response to peptides, namely, subjects 2, 4, 5, 8, 9, 12,15, 19, and 22, had in circulation DN T cells that recognized CD4 megapools. Two subjects, 14 and 16, whose single positive CD4+ and CD8+ T cells did not respond to peptides, showed a great DN response to CD4 and CD8 megapools (Figure 3, panel A).

CCR6 was also expressed on DN T cells that recognized CD4 and CD8 peptide pools, suggesting their possible homing to endothelial sites or the gut (Figure 3, panel B). The expression of programmed cell death protein-1 (PD1) correlated with the expression of the IL-2 receptor CD25, therefore suggesting that these DN T cells are activated rather than exhausted (Figure 3, panel C). It also appeared that DN T cells did not become CD4+ CD8+ double positive (DP) as the DP T cells in circulation were very few and did not respond to SARS-CoV-2 peptides (Supplementary Figure S2, panel A and B).

### 2.4. Enumeration and SARS-CoV-2 Response by Vβ21.3 T Cells

**Figure 4:**
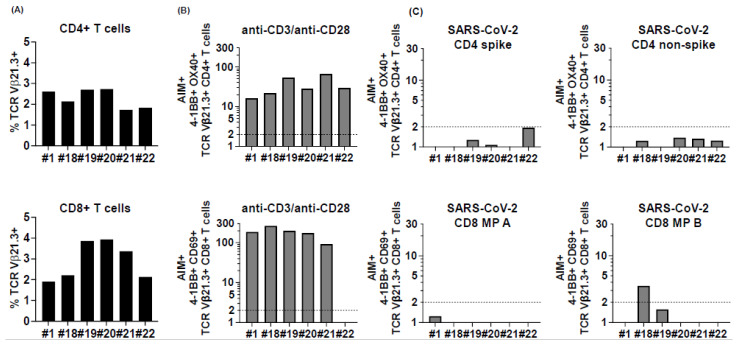
TCR Vβ21.3+ CD4+ and CD8+ T cell responses to peptide megapools derived from SARS-CoV-2 in subacute MIS-C subjects. CD4+ and CD8+ T cells from 6 (1 and 18–22) of the 22 MIS-C subjects were studied for their TCR Vβ21.3 usage and the TCR Vβ21.3+ CD4+ and CD8+ T cell responses to SARS-CoV-2. (**A**) TCR Vβ21.3 usage of CD4+ (top panel) and CD8+ (bottom panel) T cells under an unstimulated condition. An amount of 1.7%–2.7% of CD4+ cells and 1.9%–3.9% of CD8+ T cells were TCR Vβ21.3+ among the 6 subjects studied. (**B**) TCR Vβ21.3+ CD4+ (top panel) and CD8+ (bottom panel) T cell activations in response to anti-CD3/anti-CD28, SARS-CoV-2 CD4 spike and non-spike, and SARS-CoV-2 CD8 MP A and B peptide megapool stimulations. SIs of AIM+ TCR Vβ21.3+ CD4+ and CD8+ T cells from each individual subject are shown. TCR Vβ21.3+ CD4+ and CD8+ T cells from all the subjects responded to anti-CD3/anti-CD28 stimulation except for 1 subject. (**C**). The majority of the TCR Vβ21.3+ CD4+ and CD8+ T cells showed no detectable response to any of the SARS-CoV-2 peptide megapools; however, TCR Vβ21.3+ CD8+ from subject 18 showed an SI > 2 in response to SARS-CoV-2 CD8 MP B stimulation.

In an effort to understand MIS-C pathogenesis, a specific T cell receptor β chain, Vβ21.3, has been linked to the inflammatory process [11,12]. We studied the SARS-CoV-2 response by Vb21.3 CD4+ and CD8+ T cells in 6 MIS-C subjects (1,18, 19, 20, 21, 22) and confirmed previous observations that this TcR rearrangement is relevant in MIS-C with this receptor expressed in up to 2.7% of the CD4+ T cells and 3.9% of the CD8+ T cells (Figure 4, panel A and Supplementary Figure S3). Vβ21.3 T cells responded well to anti-CD3/anti-CD28 stimulation (Figure 4, panel B), but only 1 of 6 subjects (18) had CD8+ T cells that recognized one of the CD8 peptide megapools (Figure 4, panel C). Vβ21.3 CD4+ and CD8+ T cells expressed very low levels of CCR6, suggesting that they do not home to the vessels or the gut.

### 2.5. Immune Phenotype of the Antigen Presenting Cells in MIS-C

**Figure 5:**
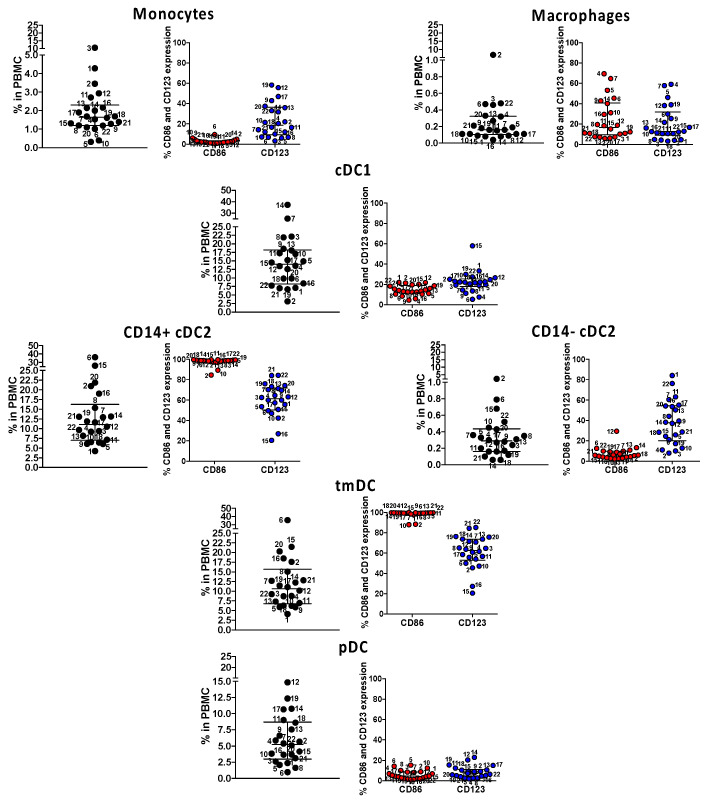
Immune phenotyping of circulating innate antigen presenting cells (APC) in subacute MIS-C subjects. We enumerated APC populations with a combination of monoclonal antibodies in all the 22 MIS-C subjects studied for SARS-CoV-2 T cell responses in their subacute phase by staining the same PBMC preparations. Monocytes were defined as CD14+ CD11c− CD11b−; macrophages were defined as CD14+ CD11c− CD11b+; myeloid cDC1 were defined as CD11c+ CD11b− CD14−; myeloid cDC2 (CD14+ or CD14−) CD11c+ CD11b+, tmDC were defined as CD11c+ CD11b+ CD4+ ILT-4+; and pDC was defined as CD14− CD11c− CD11b− CD123+. The activation/maturation markers CD86 (red symbols) and CD123 (blue symbols) are shown next to the population. Symbols represent data derived from each individual subject. Median ± interquartile ranges are indicated in the figure. CD14+ cDC2 and CD11c+ CD11b+ CD14+ CD4+ ILT-4+, functionally tolerogenic, were the most activated, as shown by the expression of CD86 and CD123.

Next, we enumerated and determined the maturation/activation state of monocytes, macrophages, and myeloid dendritic cells, including cDC1, CD14+ cDC2, CD14−, cDC2, pediatric CD4+ ILT-4+ tolerogenic DC (tmDC), and plasmacytoid DC (pDC), in the 22 subjects studied for SARS-CoV-2-specific T cell responses. CD4+ ILT-4+ tmDC is important in controlling pediatric immune homeostasis [14,15]. In MIS-C, the status of the innate compartment and the extent of the activation of tolerogenic CD14+ cDC2 and tmDC could have played a significant role in the disease pathogenesis. The results, shown in Figure 5, revealed that CD14+ cDC2 and tmDC, both suppressive myeloid lineages, were numerous in circulation and up to 100% CD123+ and 80% CD86+, suggesting both maturity and activation. All the other lineages were within physiological ranges, including pDC, important in the innate response to viruses.

### 2.6. Expansion of CD4+ Th Cells and T Cell Memory in MIS-C Studied 6–14 Months after MIS-C Onset

**Figure 6:**
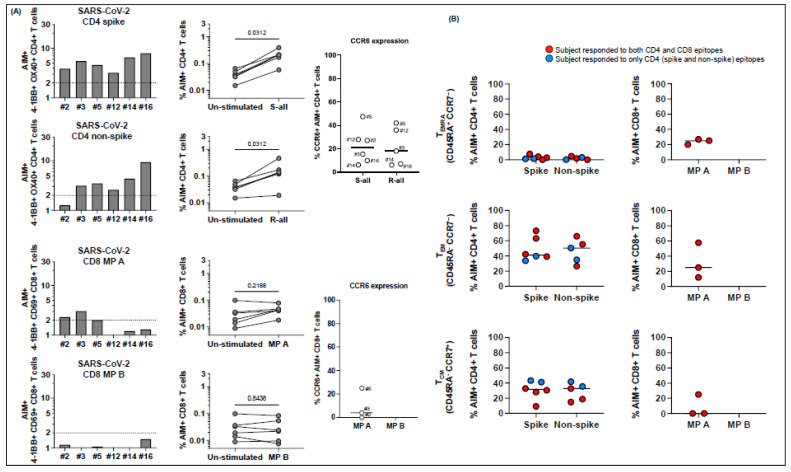

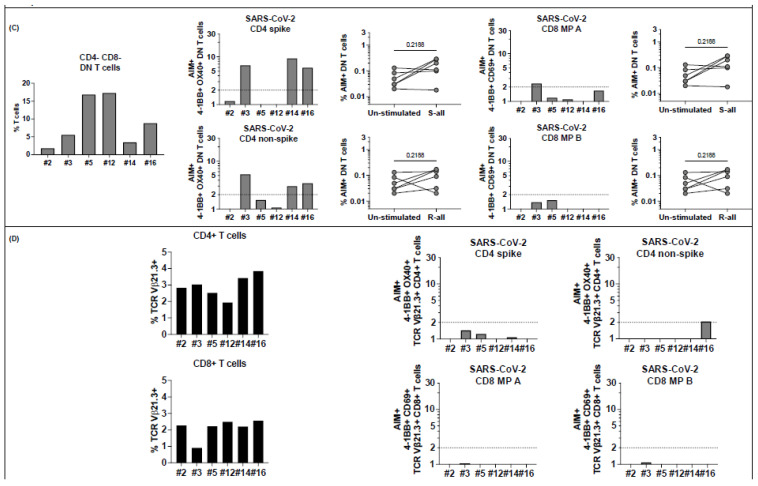
T cell responses to peptide megapools derived from SARS-CoV-2 in MIS-C subjects healthy 6-14 months after the disease onset. Six MIS-C subjects (2, 3, 5, 12, 14, and 16) studied at their subacute phase were also studied 6–14 months later. Canonical CD4+ and CD8+, CD4− CD8− DN, and TCR Vβ21.3+ CD4+ and CD8+ T cell responses to SARS-CoV-2 peptide megapools were studied. The SI of AIM+ T cells from each individual subject was calculated to study the T cell responses. (**A**) CD4+ and CD8+ T cell responses to peptide megapools and their CCR6 expressions. Three subjects (2, 3, and 5) of the 6 subjects showed concurrent CD4+ and CD8+ T cell responses, and the other 3 follow-up MIS-C subjects (12, 14, and 16) showed only CD4+ T cell responses to SARS-CoV-2 (both CD4 spike (S) and nonspike (R) megapools). (**B**) Memory phenotypes of AIM+ CD4+ (left panels) and CD8+ (right panels) T cells. Red circles: subjects responding to both CD4 and CD8 epitopes (subjects 2, 3, and 5); blue circles: subjects responding to both CD4 spike and nonspike epitopes (subjects 12, 14, and 16). AIM+ CD4+ T cells showed a similar level of TEMRA, TEM, and TCM at follow-up visit compared with subacute MIS-C subjects (*p* > 0.05). AIM+ CD8+ T cells showed a slight increase in TEM (*p* > 0.05) and a similar level of TEMRA and TCM. (**C**) Percentage of DN T cells (left panel) and their responses to SARS-CoV-2 peptide megapools (right panels). Percentages of DN T cells were decreased in 5 subjects (2, 3, 5, 12, and 14) compared with subacute MIS-C subjects. One subject (3) showed DN T cell responses to SARS-CoV-2 CD4 and CD8 peptide megapools, and 2 subjects (14 and 16) showed DN T cell responses to SARS-CoV-2 CD4 spike and nonspike peptide megapools. (**D**) Percentage of CD4+ and CD8+ TcR Vβ21.3 (black bars, left panels). An amount of 2.0–3.9% of CD4+ T cells and 0.9–2.6% of CD8+ T cells were TCR Vβ21.3+ among the 6 subjects studied. TCR Vβ21.3+ CD4+ T cells in 1 subject (16) showed a minor response to a SARS-CoV-2 CD4 nonspike peptide megapool. Each symbol represents an individual subject. Comparisons of the percentage of AIM+ T cells between unstimulated control and peptide megapool-stimulated cell cultures were tested by the Wilcoxon signed-rank test. Comparisons of the memory phenotypes of AIM+ T cells between subacute and follow-up visit were tested by the Mann–Whitney U test.

Of the 22 MIS-C subjects, 6 (27%) (2, 3, 5, 12, 14, and 16) were studied for their SARS-CoV-2 T cell responses in their subacute phase and again 6–14 months later. The magnitude of the CD4+ Th cell responses to the spike proteins increased in 4 subjects (5, 12, 14, and 16) (Figure 6, panel A). T cells from subjects 5, 12, 14, and 16 that did not respond to nonspike CD4 megapools in the subacute phase of MIS-C showed a response in the follow-up visit, suggesting epitope spreading over time (Figure 6, panel A). Epitope spreading included CD8+ T cell responses in subjects 2 and 3 at the follow-up visit (Figure 6, panel A). The distribution of CCR6 on CD4+ and CD8+ T cells was similar to what we observed in the subacute phase in all the 6 subjects studied (Figure 6, panel A). Within the development of T cell memory, TEMRA cells were absent in the CD4 compartment but numerous within CD8+ T cells (Figure 6, panel B). CD4+ TEM increased from the subacute phase, showing increased T cell memory to spike and nonspike proteins (Figure 6, panel B). DN T cells were still numerous in circulation, and as in the subacute phase, subjects 3, 14, and 16 recognized SARS-CoV-2 CD4 peptides (Figure 6, panel C). Interestingly, Vβ21.3 T cells were still present several months (2.0–3.9% of CD4+ T cells; 0.9–2.6% of CD8+ T cells) after the acute illness and, as in the subacute phase, did not respond to SARS-CoV-2 peptides (Figure 6, panel D).

## 3. Discussion

This study addresses the T cell recognition of SARS-CoV-2 epitopes and the innate immune phenotype in MIS-C patients studied between March 2021 and March 2022. The magnitude of the T cell response in our MIS-C cohort possibly infected by SARS-CoV-2 variants (Delta and Omicron) later in the pandemic was similar to the CD4+ Th response and the CD8+ CTL response that we reported in the first year of the pandemic [10]. Several subjects had only an antiviral CD4+ Th response, which in MIS-C seems to be unique, even though CD8+ CTLs were thought to be pathogenic by some investigators.

In fact, a correlation has been suggested between MIS-C disease severity and class I HLA A2, HLA B35, and HLA C4 that could present superantigens derived from the SARS-CoV-2 spike glycoproteins to specific oligoclonal TcRs carrying the Vβ21.3 chain, encoded by the *TRBV11-2* gene [11]. Other authors found an oligoclonal expansion of Vβ21.3 T cells, which were also believed to be pathogenic [16]. We found numerous Vβ21.3 CD4+ and CD8+ T cells in our MIS-C cohort, but only in 1 subject CD8+ T cells recognized SARS-CoV-2 peptides, suggesting that the specificity is skewed to a different antigen or autoantigen. Evidence that healthy children studied 6 to 14 months after MIS-C still have numerous Vβ21.3 CD4+ and CD8+ T cells in circulation suggests that it is unlikely that these T cells are pathogenic but rather a characteristic of the T cell repertoire in these children.

Our data on the SARS-CoV-2-specific T cell response did not indicate a defect in the anti-viral-specific T cell repertoire, nor did we observe differences in the pattern of Th and CTL expansion in MIS-C subjects enrolled in 2020 [10], compared with MIS-C subjects enrolled in 2021 and 2022 and likely exposed to different variants of SARS-CoV-2. In this study, a high percentage of T cells, especially CD4+ T cells, expressed CCR6, which suggests trafficking to the endothelium, lungs, and gut that express CXCL20, the ligand for CCR6.

Consistent with our previous study, differences in the development of SARS-CoV-2-specific T cell memory were observed with numerous effector and central memory T cells within the Th but not CTL compartment. Terminally differentiated effector T cells were abundant within the SARS-CoV-2-specific CD8+ but not CD4+ T cells, which may suggest a different timing of memory development.

A novel observation in this work is the antiviral function of DN T cells that recognize SARS-CoV-2 CD4+ and CD8+ peptide epitopes. DN T cells in the thymus become CD4+ CD8+ double positive (DP) to then mature as CD4+ or CD8+ single-positive, depending on the recognition of peptides presented by either MHC class II or class I molecules [17]. In adults, DN T cells are well described in systemic autoimmunity and via lymphokine secretion they contribute to the inflammatory milieu [18]. The DN T cells that we describe here are phenotypically different and recognize not only longer CD4 peptides but also short viral peptides contained in the CD8 peptide pools, suggesting recognition in association with both MHC class I and class II. Therefore, these DN T cells are already committed despite the absence of CD4 or CD8 coreceptors. Additionally, exhausted T cells may downregulate the CD4+ or CD8+ coreceptors under control of FAS signaling [19], but the expression of the IL-2 receptor CD25 that we reported associated with PD1 indicate functional T cell activation. In MIS-C, DN T cells in circulation could compensate for the severe lymphopenia in these patients; alternatively, DN T cells could have contributed to the inflammation by recognizing autoantigens. Evidence that DN T cells are virus specific and actually numerous in children that appeared to be nonresponders with canonical CD4+ and CD8+ T cells suggests that they are a mature T cell subset that participate in the antiviral response.

A sharp difference in MIS-C children enrolled in 2021–2022 versus children enrolled during the first year of the COVID-19 pandemic is the immune phenotype of the innate compartment: cDC2 and tmDC, tolerogenic, anti-inflammatory lineages, were low in our previous study [10] but numerous in this cohort.

We recognize both strengths and limitations to our study. We present a comprehensive characterization of the T cell response to SARS-CoV-2 and a detailed analysis of the innate APC compartment and enumeration and characterization of a specific TcR rearrangement that has been previously implicated in the inflammatory process. The novelty of finding numerous DN T cells, fully competent in responding to the virus and in the absence of DP T cells, undermines the paradigm of T cell selection, suggesting that in the absence of CD4+ and CD8+ coreceptors, the TcR is fully committed. A limitation is the small number of subjects studied and the lack of access to tissues to better define T cell homing and trafficking.

In summary, SARS-CoV-2-infected children who subsequently developed MIS-C showed a prevalent CD4+ Th response to the virus that reflected on the T cell memory phenotype where TEM and TCM cells were predominantly CD4, numerous DN T cells, and the activation of tolerogenic DC as CD14+ cDC2 and CD14+ CD11c+ CD11b+ ILT-4+ tmDC important to downsize the inflammation. Vb21.3+ T cells were detectable but did not recognize SARS-CoV-2 T cell epitopes. Over time, T cell epitope spreading occurred, as shown by the increased magnitude of the T cell responses and the expansion of T cell specificities in the late convalescent phase.

## 4. Materials and Methods

### 4.1. Study Populations

The study protocol for MIS-C and KD subjects was approved by the Institutional Review Board at the University of California San Diego (IRB #140220). Subjects were enrolled at Rady Children’s Hospital, San Diego, following written parental informed consent and patient assent as appropriate. Twenty-two MIS-C subjects, 17 males and 5 females, aged 1.8 to 15 years were enrolled in the study from March 2021 to March 2022, 15–52 days after MIS-C onset to study SARS-CoV-2 T cell responses and to assess their innate immune phenotype. MIS-C patients’ clinical and laboratory information at the time of hospital admission is described in Table 1. SARS-CoV-2 exposure was determined by PCR and antibody measurement. Blood samples were collected following intravenous immunoglobulin (IVIG) and other anti-inflammatory treatments 15–52 days after fever onset. Six of these subjects (2, 3, 5, 12, 14, and 16; Table 1) were studied at their follow-up visit 6–14 months after resolution of acute MIS-C symptoms.

### 4.2. Peptide Megapools

Two SARS-CoV-2 CD4 megapools and two SARS-CoV-2 CD8 megapools were used to study the CD4+ and CD8+ T cell responses to SARS-CoV-2 in 22 MIS-C subjects. The megapools were designed based on the reference genomic sequence of Wuhan-Hu-1 SARS-CoV-2 isolate (GenBank ID: MN908947), as described and validated in acute and convalescent SARS-CoV-2-infected patients and unexposed healthy subjects [7,8]. The SARS-CoV-2 CD4 spike megapool contains 253 15-amino-acid-long peptides overlapping by 10 amino acids and spanning the entire spike protein. The SARS-CoV-2 CD4 nonspike megapool contains 221 15-mers predicted HLA class II epitopes derived from the remainder (nonspike) of the SARS-CoV-2 proteome. The two SARS-CoV-2 CD8 megapools contain a total of 628 peptides (314 in each megapool), predicted to bind 12 HLA A and B most frequent alleles in the general human population (A*01:01, A*02:01, A*03:01, A*11:01, A*23:01, A*24:02, B*07:02, B*08:01, B*35:01, B*40:01, B*44:02, and B*44:03). Peptides were synthesized as crude material (T.C. Laboratories, San Diego, CA, USA), resuspended in DMSO, pooled according to megapool design, and relyophilized (Carrasco Pro, 2015 #274).

### 4.3. Activation-Induced Markers (AIM) Assay and Enumeration of Vβ21.3 T Cells

Peripheral blood mononuclear cells (PBMCs) were separated from heparinized whole blood from MIS-C convalescent SARS-CoV-2 infected and KD subjects by Ficoll-Hypaque density centrifugation and frozen in liquid nitrogen. After thawing, 1 × 10^6^ cells were stimulated in 96-well U-bottom plates with 1 μg/mL of different peptide megapools. PBMCs cultured with 0.1% DMSO, the same concentration of DMSO (solvent) in the megapool-stimulated cultures, served as unstimulated controls. After 24 h, cell cultures were harvested and stained with monoclonal antibodies and analyzed by flow cytometry (Cosarizza, 2019 #296) to study T cell activation, CCR6 expression, and effector and memory phenotypes: anti-CD3-AF700 (clone OKT3, mouse IgG2aκ, BioLegend, 8999 Biolegend Way, San Diego, CA 92121), anti-CD4-BV605 (clone RPA-T4, mouse IgG1κ, BD Biosciences, 10975 Torreyana Rd, San Diego, CA 92121, USA), anti-CD8-BV650 (RPA-T8, mouse IgG1κ, BioLegend), anti-4-1BB-allophycocyanin (clone 4B4-1, mouse IgG1κ, BioLegend), anti-OX40-PE/Cy7 (clone Ber-ACT35, mouse IgG1κ, BioLegend), anti-CD69-PE (clone FN50, mouse IgG1κ, BD Biosciences), anti-CCR6-PerCp/Cy5.5 (clone 11A9, mouse IgG1κ, BD Biosciences), anti-CD45RA-BV421 (clone HI100, mouse IgG1κ, BioLegend), and anti-CCR7-FITC (clone G043H7, mouse IgG2aκ, BioLegend). Anti-TCR Vβ21.3-PE/Vio615 (clone REA894), recombinant human IgG1, Milteny, 6125 Cornerstone Court East San Diego, CA 92121) was used to define CD4+ and CD8+ V, 21.3 T cells.

Data were recorded on LSRFortessa X-20 (BD Biosciences) and analyzed with FlowJo software version 10 (Tree Star). Isotype controls for each antibody were tested and showed no staining.

Antigen-specific responses were determined by the expression of T cell activation-induced cell marker (AIM) assay by measuring the coexpression of 4-1BB and OX40, two TNF family member costimulatory molecules upregulated following T cell receptor signaling on CD4+ T cells, and by measuring the coexpression of 4-1BB and CD69 (adhesion molecule involved in lymphocyte homing and trafficking) on CD8+ T cells [7]. The expression of the chemokine receptor CCR6 on AIM+ CD4+ and CD8+ T cells was also analyzed. Terminally differentiated effector T cells (TEMRA, CD45RA+ CCR7−), effector memory T cells (TEM, CD45RA− CCR7−), and central memory T cells (TCM, CD45RA− CCR7+) were enumerated on AIM+ CD4+ and CD8+ T cells.

### 4.4. Immune Phenotyping of Myeloid Antigen-Presenting Cells (APC)

Innate myeloid cells were defined by surface markers by staining with monoclonal antibodies and analyzed by flow cytometry gating on specific populations: antihuman CD11c-allophycocyanin, clone B-ly6, mouse IgG1κ; antihuman CD11b-allophycocyanin/Cy7, clone ICRF44, mouse IgG1κ; antihuman CD14-PE/Cy7, clone M5E2, mouse IgG2aκ (BD Biosciences); antihuman BDCA-1-PE/Dazzle594, clone L161, mouse IgG1κ (BioLegend); antihuman ILT-4-PerCp/eF710, clone 42D1, rat IgG2aκ (eBioscience); antihuman CD4-AF700, clone RPA-T4, mouse IgG1κ (BD Biosciences); and antihuman CD16-BV605, clone B73.1, mouse IgG1κ. The activation/maturation of the innate immune cells that present antigen to T cells was defined by the expression of CD86 by using antihuman-CD86 FITC, clone FUN-1, mouse IgG1κ (BD Biosciences). Data were acquired on BD CANTO II and analyzed with FlowJo software version 10 (Tree Star).

### 4.5. Statistical Analysis

Data were analyzed using Prism software version 9.0 (GraphPad Software). To compare the percentage of AIM+ T cells in the unstimulated control and peptide stimulation, data obtained from each peptide megapool-stimulated culture and unstimulated controls in the individual cohort were tested using nonparametric paired tests. A *p*-value < 0.05 was considered significant.

## Figures and Tables

**Table 1:** Demographic and clinical characteristics of MIS-C patients.

Variable *	MIS-C (n = 22)
**Demographic information**	
Age, yrs	9.1 (5.3–12.0)
Male, n (%)	17 (77)
Female, n (%)	5 (23)
Ethnicity, n (%)	
Asian	1 (5)
African American	2 (9)
White	2 (9)
>2 races or other	6 (27)
Hispanic	11 (50)
**Cardiac data and clinical course**	
Zmax †	1.5 (0.8–2.2)
Lowest LV Ejection Fraction	56.5 (47.8–60.8)
ICU admission, n (%)	9 (41)
SARS-CoV-2 antibody positive, n (%)	22 (100)
**Clinical laboratory data**	
Illness day of Sample collection ^‡^	5 (4–6)
WBC, 10^3^/mL	9.9 (6.1–14.5)
Polys, %	71 (56–81)
Bands, %	17 (8–25)
Lymphocytes, %	10 (6–12)
ANC, /mL	8391 (4675–13,230)
ZHgb	−1.8 (−2.5 to −1.0)
PLT, ×10^3^/mm^3^	148 (100–201)
ESR, mm/h ^§^	35 (25–57)
CRP, mg/dL	22 (17–29)
ALT, IU/L	37 (26–51)
Sodium, mmol/L	132 (130–134)
BNP, pg/mL ^¶^	215 (40–644)
**Treatment**	
Intravenous immunoglobulin	21 (95)
Steroids	15 (68)
Infliximab	10 (45)
Anakinra	10 (45)

*: median (Interquartile range (IQR)) unless specified. †: Maximum Z score (internal diameter normalized for body surface area) for the right and left anterior descending coronary arteries. ‡: Illness Day 1 = first day of fever. §: ESR was available from 20 patients. ¶: BNP was available from 21 patients. LV: left ventricle, WBC: white blood cell count, ANC: absolute neutrophil count, ABC: absolute band count, ZHgb: hemoglobin concentration normalized for age, ESR: erythrocyte sedimentation rate, CRP: C-reactive protein, ALT: Alanine aminotransferase, BNP: B-type natriuretic peptide.

## Data Availability

The data that support the findings of this study are available from the corresponding author upon reasonable request.

## Supplementary Materials

The following supporting information can be downloaded at: https://www.mdpi.com/article/10.3390/ijms23137219/s1.
