# Supplementary material for: T Cells in Multisystem Inflammatory Syndrome in Children (MIS-C) Have a Predominant CD4+ T Helper Response to SARS-CoV-2 Peptides and Numerous Virus-Specific CD4− CD8− Double-Negative T Cells
Source: Int J Mol Sci. 2022 Jun 29;23(13):7219. doi: 10.3390/ijms23137219 (PMC9266459; doi:10.3390/ijms23137219)
Supplement: Supplementary file 1 [file ijms-23-07219-s001.zip › ijms-1788211-supplementary.pdf]

Supplementary materials

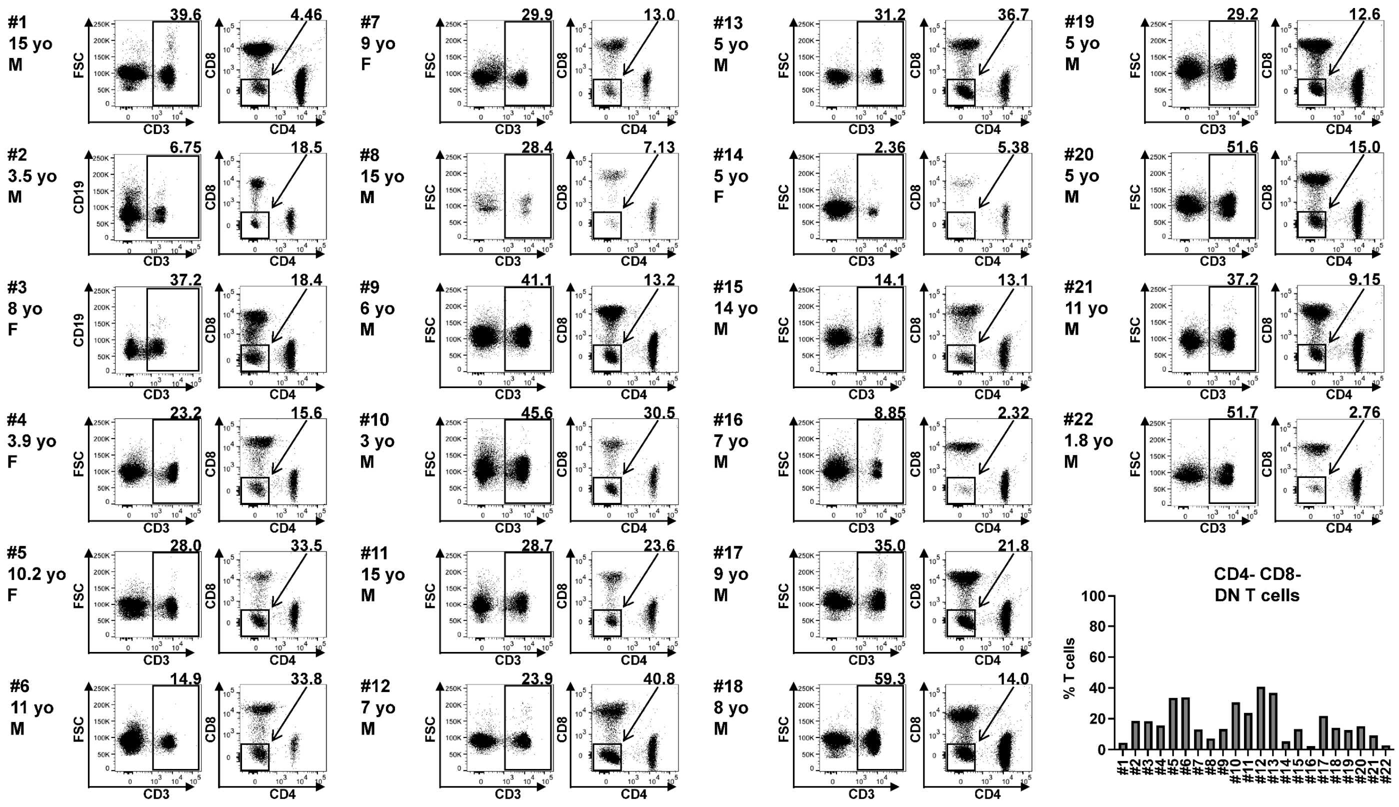

Supplementary Figure S1. CD4− CD8− DN T cells in subacute MIS-C subjects. FACS plots showing the gating of CD4− CD8− DN T cells. Percentage of the gated population is indicated at the top right corner of the plots. DN T cells were numerous (median 14.5%; Q1 – Q3: 8.65 – 25.3%) in the total T cell population in subacute MIS-C subjects.

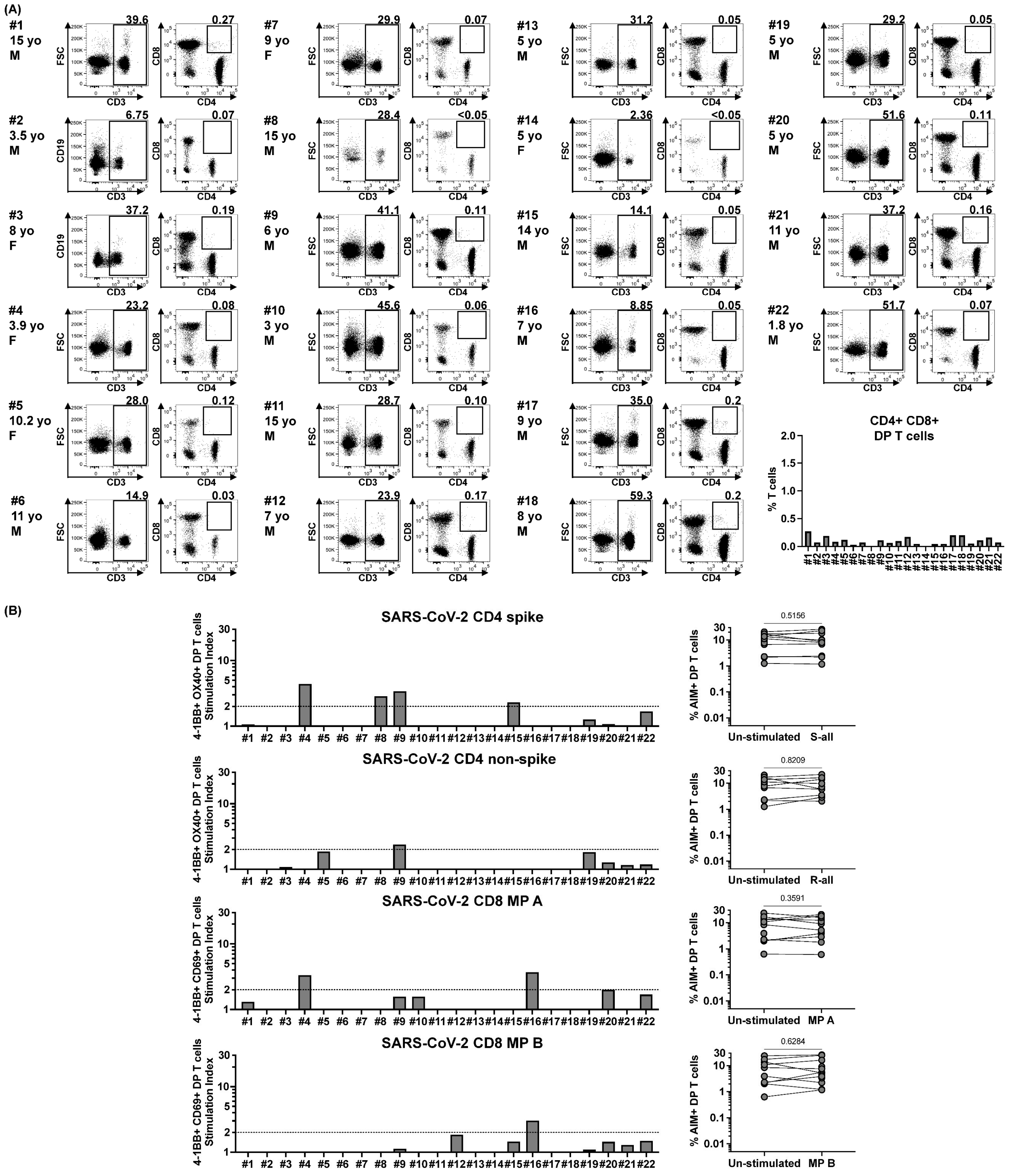

Supplementary Figure S2. CD4+ CD8+ double positive (DP) T cells in subacute MIS-C subjects.

(A) FACS plots showing the gating of CD4+ CD8+ DP T cells. Percentage of the gated population is indicated at the top right corner of the plots. DP T cells were detectable in a low range (median

0.0785%; Q1 – Q3: 0.0478 – 0.163%) in the total T cell population in subacute MIS-C subjects. **(B)** DP T cell activation in response to SARS-CoV-2 peptide megapool stimulations. SI of AIM+ DP T cells from each individual subject were shown. One (#4) of the 22 subjects showed DP T cell responses to SARS-CoV-2 CD4 and CD8 peptide megapools concurrently. Three (#8, 9, 15) and one (#16) of the 22 subjects showed response to SARS-CoV-2 CD4 and CD8 peptide megapools, respectively. Seventeen (#1 – 3, 5 – 7, 10 – 14, #17 – 22) showed no measurable response from DP T cells. Each symbol represents the data derived from individual subject. Comparisons of the percentage of AIM+ T cells between un-stimulated control and peptide megapool-stimulated cell cultures were tested by Wilcoxon signed rank test.

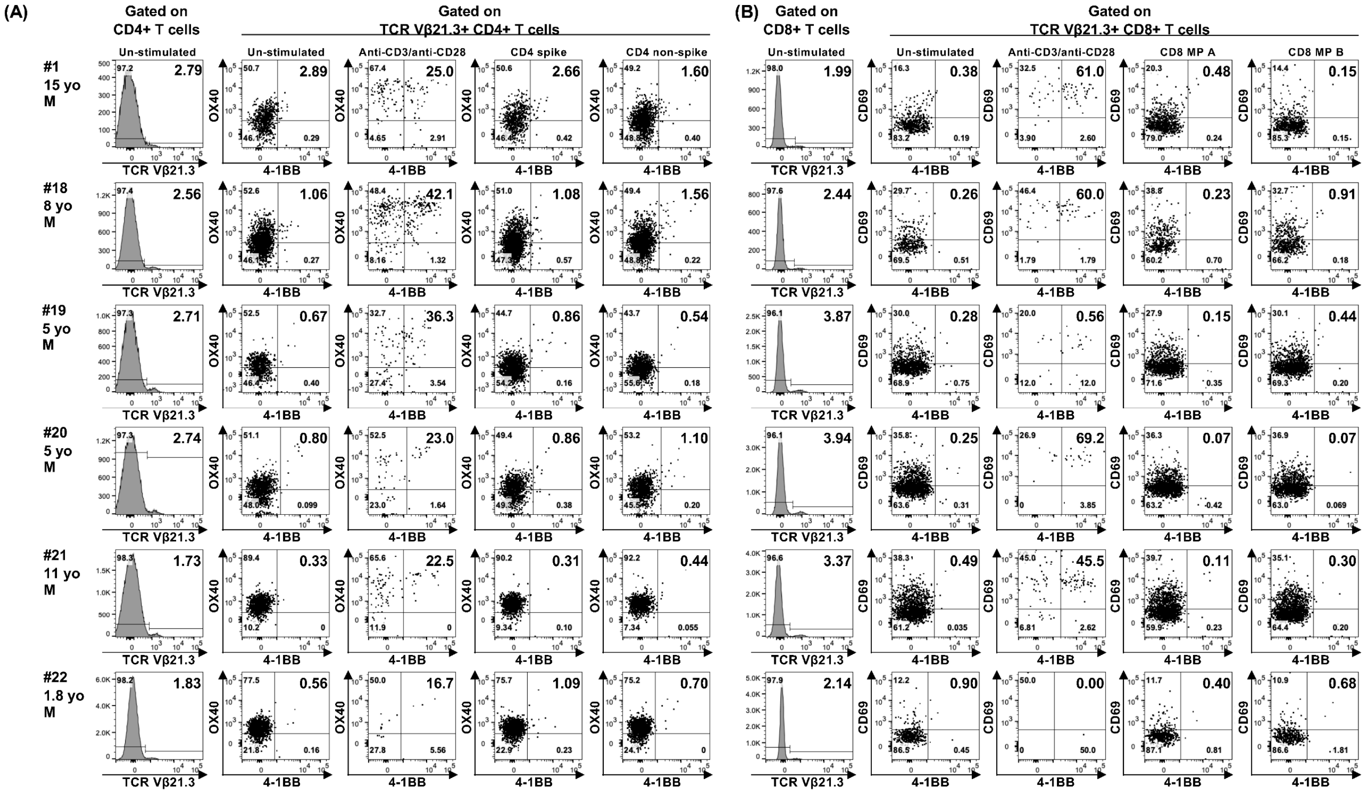

Supplementary Figure S3. TCR Vβ21.3+ CD4+ and CD8+ T cells in subacute MIS-C subjects. FACS plots showing the percentage of TCR Vβ21.3+ under **(A)** CD4+ and **(B)** CD8+ T cells and their expression of AIM under different stimulatory conditions. Percentage of the gated population is indicated at the top right corner of the plots.

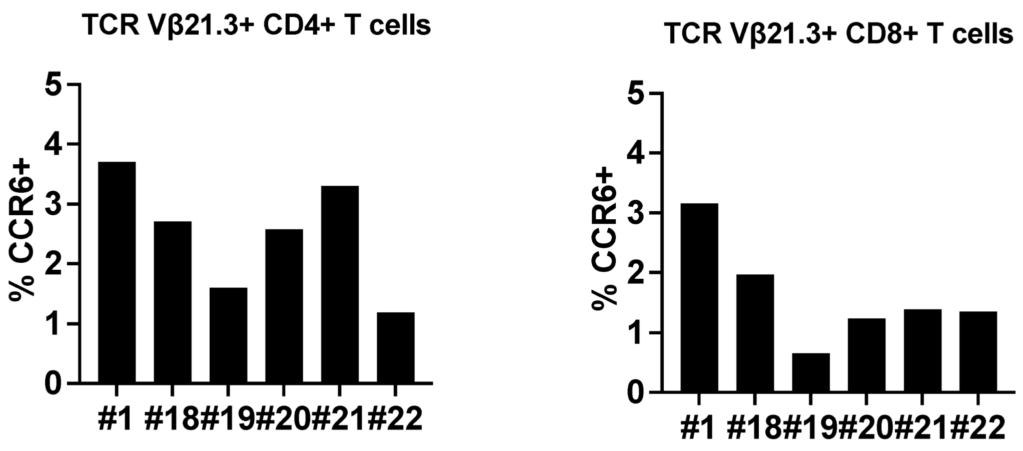

Supplementary Figure S4. CCR6 expression on Vβ 21.3 CD4+ and CD8+ T cells

The expression of the chemokine receptor CCR6 has been studied on Vβ 21.3 T cells by flow cytometry and found on the cell surface in a small percent of CD4+ T cells (left panel) and CD8+ T cells (right panel).
